# Supplementary material for: Evaluation of the Laguerre–Gaussian mode purity produced by three-dimensional-printed microwave spiral phase plates
Source: R Soc Open Sci. 2020 Jul 22;7(7):200493. doi: 10.1098/rsos.200493 (PMC7428225; doi:10.1098/rsos.200493)
Supplement: Figure S1;Figure S2;Figure S3 [file rsos200493supp1.pdf]

Supplemental Materials

Evaluation of the Laguerre-Gaussian Mode Purity Produced by 3D-Printed Microwave Spiral Phase Plates

D. Isakov[1,2], Y. Wu[2], B. Allen[3], P. S. Grant[2], C. J. Stevens[3], and G. Gibbons[1]

[1] WMG, University of Warwick, Coventry CV4 7AL, UK
[2] Department of Materials, University of Oxford, Oxford OX1 3PH, UK
[3] Department of Engineering Science, University of Oxford, Parks Road, Oxford, OX1 3PJ, UK

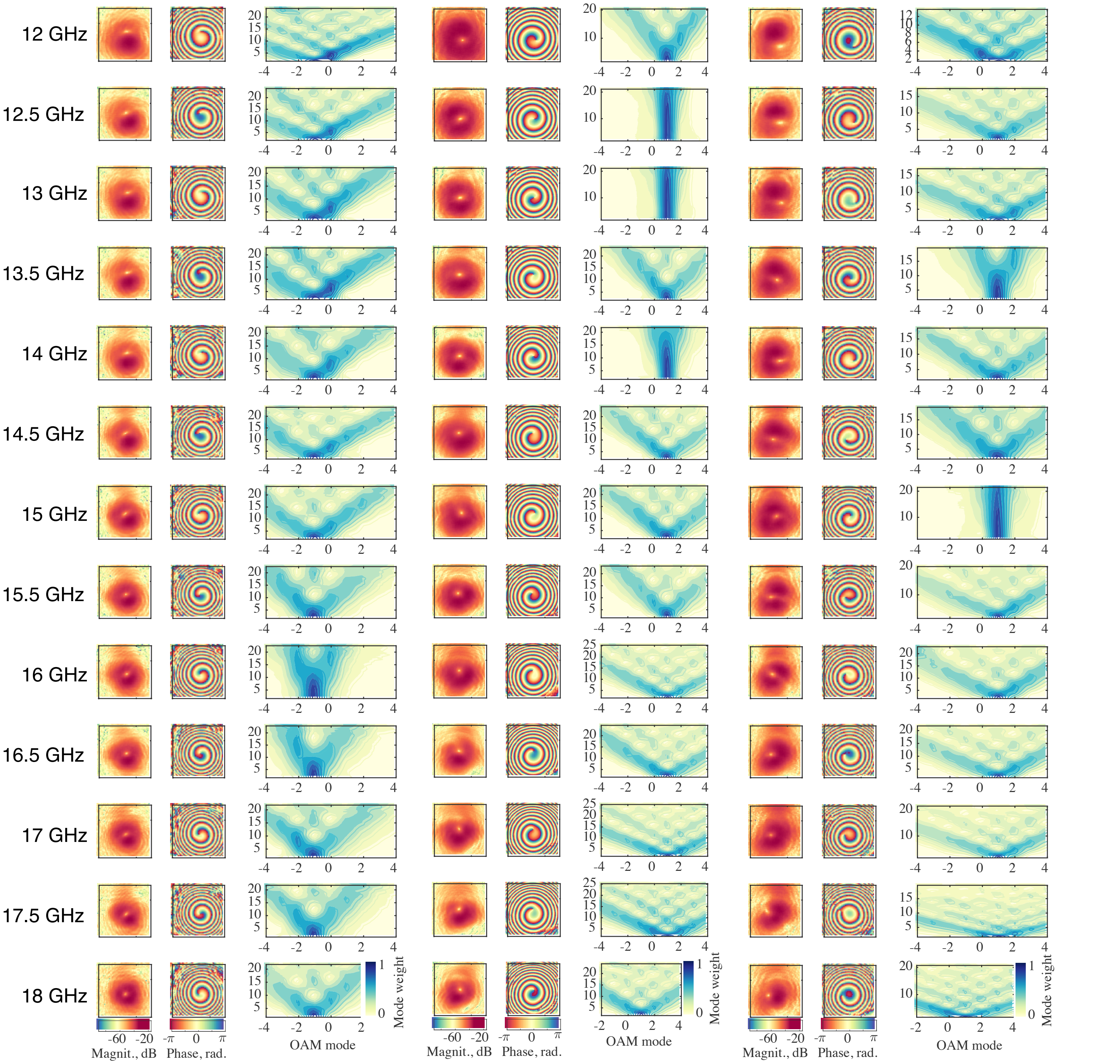

Figure S1. Measurements of the radiation signal passed through an Smooth SPP (left), Staircase SPP (middle) and GRIN SPP (right) in the X-Y plane of area 700 × 700 mm² perpendicular to the propagation vector. Columns corresponds to S_{21} amplitude profile, S_{21} phase profile, and OAM mode spectrum.

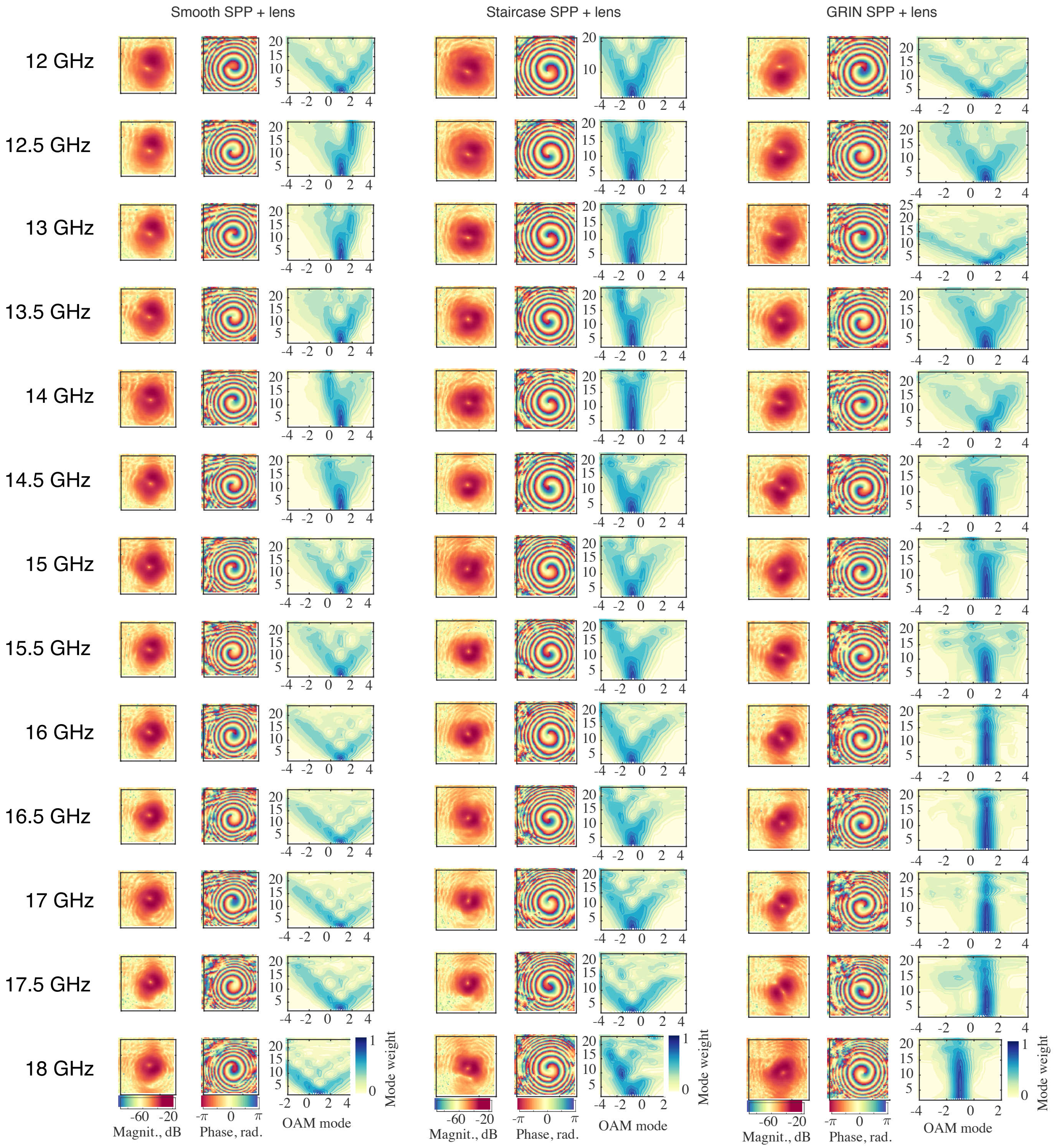

Figure S2. Measurements of the radiation signal passed through transmission system coupled with SPP and lens. The order is the same as in Fig. S1: Smooth SPP (left), Staircase SPP (middle) and GRIN SPP (right). Columns corresponds to S_{21} amplitude profile, S_{21} phase profile, and OAM mode spectrum.

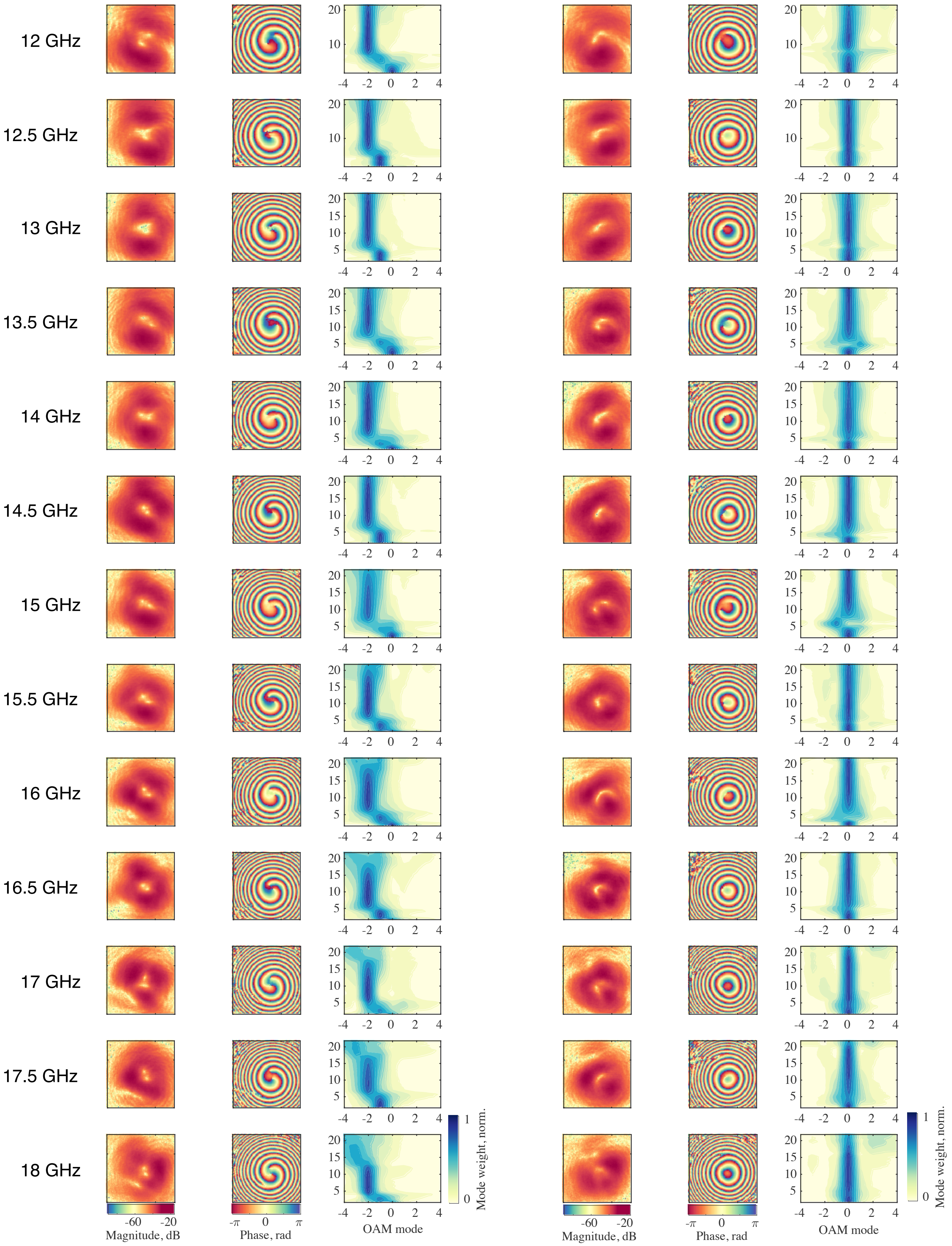

Figure S3. Amplitude, phase and OAM mode power spectrum for $l_2 = 1 + 1$ multiplexing (left) and $l_0 = 1 - 1$ (de)mmultiplexing (right) of the OAM mode 1 signals.
